# Three-dimensional kinematics of shoulder laxity examination and the relationship to clinical interpretation^*^

**DOI:** 10.1080/23335432.2017.1372217

**Published:** 2017-12-15

**Authors:** Justin L. Staker, Amy E. Lelwica, Paula M. Ludewig, Jonathan P. Braman

**Affiliations:** a Division of Rehabilitation Science, Department of Rehabilitation Medicine, Medical School, The University of Minnesota Medical School, Minneapolis, MN, USA; b Department of Orthopaedic Surgery, University of Minnesota, Minneapolis, MN, USA; c Division of Physical Therapy, Department of Rehabilitation Medicine, Medical School, The University of Minnesota Medical School, Minneapolis, MN, USA

**Keywords:** Shoulder biomechanics, laxity test, reliability, validity, examination

## Abstract

Understanding clinical test kinematics improves utility of exam techniques. The purposes of this study were as follows: (1) determine inter-examiner repeatability of translation magnitude for the Anterior/Posterior Drawer and Sulcus shoulder laxity tests; (2) describe the relationships between glenohumeral joint translations and subjective grades for each laxity test; and (3) describe the relationship of overall glenohumeral joint laxity to a composite subjective score from the three laxity tests. Eleven subjects with shoulder symptomology were examined with three laxity tests. Motion was tracked with electromagnetic sensors affixed to the humerus and scapula via transcortical pins. ICCs were calculated to determine repeatability of translation magnitudes between two examiners for each test. Descriptive statistics and regression analyses were performed for comparisons of single laxity test grades with translation magnitudes and for composite subjective laxity scores and overall translation across all three tests. Inter-examiner ICCs regarding kinematic repeatability were 0.87 for Anterior Drawer, 0.84 for the Sulcus test, and not calculable for the Posterior Drawer. No linear relationships between subjective grades of individual tests and translation magnitudes were found. The relationship of overall translation with the composite subjective score from all laxity tests was *r*^2^ = 0.75 (*r* = 0.86). Clinicians from different disciplines are capable of imparting similar translations during laxity tests. Single-test subjective laxity grades demonstrate large ranges of translation between subjects for the same grade. By combining results of three laxity tests, clinicians are capable of identifying the level of overall shoulder joint laxity in patients.

## Introduction

Shoulder pain is the second most prevalent musculoskeletal complaint with a 21% point prevalence (Picavet and Schouten 2003). Despite this, reliability and validity for many shoulder clinical examination techniques have not been demonstrated (Hegedus et al. 2012). Most clinical shoulder tests are designed to elicit a sign or symptom as a result of tissue being placed under stress by the test position. Unlike the majority of clinical tests, Anterior/Posterior Drawer, and Sulcus tests are developed to quantify the magnitude of glenohumeral translations, or joint laxity, through subjective grading (Neer and Foster 1980; Gerber and Ganz 1984; Hawkins and Mohtadi 1991). The results of laxity tests are used to infer how the magnitude of observed laxity may contribute to movement abnormalities and symptomology (Bahk et al. 2007). Increased joint laxity is thought to lead to excessive and deleterious glenohumeral translations during functional movements (Neer and Foster 1980; Matsen et al. 2006; Longo et al. 2015). However, the ability of clinicians to determine the severity of joint laxity during a manual clinical exam has not been established (Tate et al. 2012; Walker et al. 2012). Therefore, construct validation of these laxity tests should assess any relationship of subjective grades to the amount of humeral head translation during testing.

Instead, shoulder laxity clinical tests have been examined through reliability studies of subjective grading systems (Levy et al. 1999; Ellenbecker et al. 2002; Tzannes et al. 2004). Inter-examiner reliability has ranged from poor to fair with studies utilizing differing rating systems, subjects, laxity test procedures, and examiner training. No studies have assessed the inter-examiner repeatability of the translations being induced during the test maneuvers. Establishing that different examiners can impart similar translations to the same patients is a prerequisite for improving inter-examiner grading reliability and clarity of diagnosis.

Studies of clinical tests should replicate scenarios under which the tests are used and on patients on which they are applied. For example, since clinicians with differing clinical disciplines and training typically perform clinical laxity tests, examination of the repeatability of imparted joint translations by examiners of differing backgrounds is needed. Additionally, clinicians commonly use clinical laxity tests across a broad range of patient presentations. The same tests may be applied to patients where instability is likely (e.g. those with unstable shoulders) and those where laxity is less likely (e.g. ‘impingement’ patients). Therefore, it is also necessary to study these tests in a population without a history of instability or dislocation but where the possibility of ‘microinstability’ may contribute to their symptoms (Jobe and Pink 1993; Ellenbecker et al. 2002; Boileau et al. 2011). Previous work examining glenohumeral laxity has utilized radiographic and ultrasound imaging to measure joint translations (Borsa et al. 2001; Borsa, Wilk, et al. 2005; Cheng et al. 2008). These studies have demonstrated good reliability and accuracy in assessing joint laxity with mechanical devices as it relates to stress/strain characteristics of the joint. No studies have tracked translations during manually imparted clinical laxity tests and studied their relationship to subjective grades of joint laxity.

Furthermore, an individual clinical test is rarely performed or interpreted in isolation of other tests. Clinical recommendations frequently emphasize the necessity of including multiple tests for the proper evaluation of shoulder conditions (Hawkins and Mohtadi 1991; Matsen et al. 2006; Rockwood et al. 2009; Hegedus et al. 2012). Additionally, there is some evidence suggesting that combining outcomes from multiple tests increases diagnostic accuracy (Wainner et al. 2003; Walsworth et al. 2008; Michener et al. 2009). No studies have examined how subjective grades from a combination of laxity tests relate to overall joint laxity. Understanding this relationship may improve the ability to diagnose distinct movement patterns and develop more effective interventions in subgroups of patients.

This study utilized three-dimensional electromagnetic sensors rigidly affixed to the humerus and scapula to precisely (Ludewig et al. 2002) measure glenohumeral translations. Bony fixation eliminates surface-based skin motion errors previously identified as up to 17% of total humeral head translation occurring during laxity tests (Harryman et al. 1992). Additionally, with rigid tracking of bone motion, measurement error due to operator technique is eliminated compared to its possibility in imaging-based tracking techniques such as ultrasonography or radiography. This tracking technique was utilized for the following study purposes; (1) determine inter-examiner, cross discipline repeatability of translation magnitude for the Anterior/Posterior Drawer and Sulcus shoulder laxity tests; (2) describe the relationships between glenohumeral joint translations and the subjective grades for each laxity test; and (3) describe the relationship of overall glenohumeral joint laxity to a composite subjective score from the three laxity tests in subjects without a history of subluxation or dislocation.

## Methods

### Subjects

This study combined data collected from 11 volunteers with atraumatic symptomatic shoulders. The subjects were recruited for a previously published study group (Ludewig et al. 2009). Subjects were included according to the criteria listed in Table 1. These were chosen to represent a clinical presentation typical for the shoulder ‘impingement’ diagnosis (Braman et al. 2014). In a heterogeneous cohort such as this subject population, identifying cases of ‘microinstability’ is considered important for treatment planning (Boileau et al. 2011; Kibler et al. 2012). Demographic data of the subjects are included in Table 2. The Institutional Review Board of the University of Minnesota approved the study protocol. Written informed consent was obtained from all participants prior to testing.

**Table 1.** Subject inclusion/exclusion criteria.

Inclusion	Exclusion
• 18–60 years of age • Shoulder pain during active shoulder motion • Current localized anterolateral shoulder pain • Pain with resisted internal or external rotation • At least two positive impingement tests: Hawkins-Kennedy, Neer or Jobe. • Visible scapula dyskinesia^a^	• Joint disease (osteoarthritis or rheumatoid arthritis) • 25% or greater reduction in glenohumeral internal or external rotation when compared to opposite shoulder • Reproduction of symptoms during cervical spine screening • Positive drop arm or apprehension tests • History of shoulder surgery, known labral tear, or known rotator cuff tear • Previous fracture of clavicle, scapula, humerus • Symptom onset following trauma • History of glenohumeral dislocation

a Scapular dyskinesia was defined as excessive medial border or inferior border prominence during raising or lowering the arm similarly described by McClure et al. (2009).

**Table 2.** Subject demographic data.

Age, years	37.8 (14.5)
Gender	6 females/5 males
Height, cm	170.0 (10.3)
Mass, kg	78.7 (10.7)
BMI	27.2 (4.5)
Handedness (right), *n*	11
Dominant side tested, *n*	9
Symptom duration, years	9.4 (7.8)
VAS (0–10)	2.5 (1.7)
DASH (0–100)	19.8 (10.8)

Note:

BMI, body mass index; *n*, number; DASH, Disability of the Arm Shoulder and Hand; VAS, visual analog scale of usual shoulder symptom pain severity.

Mean (SD).

### Instrumentation

Kinematic data were collected using the Flock of Birds mini-BIRD electromagnetic (EM) sensors (Ascension Technology, Shellburne Vermont, USA) and processed using integrated Motion Monitor software (Innovative Sports Training, Inc. Chicago, IL, USA). This configuration allowed simultaneous tracking of each sensor at a sampling rate of 100 Hz per sensor. The instrumentation static accuracy is reported to be 1.8 mm and 0.5° (Ascension Technology Corporation). We verified in our lab for this experiment that the root mean square linear static accuracy of the instrumentation was less than 1 mm compared to a calibration grid.

### Procedures

**Figure 1.**
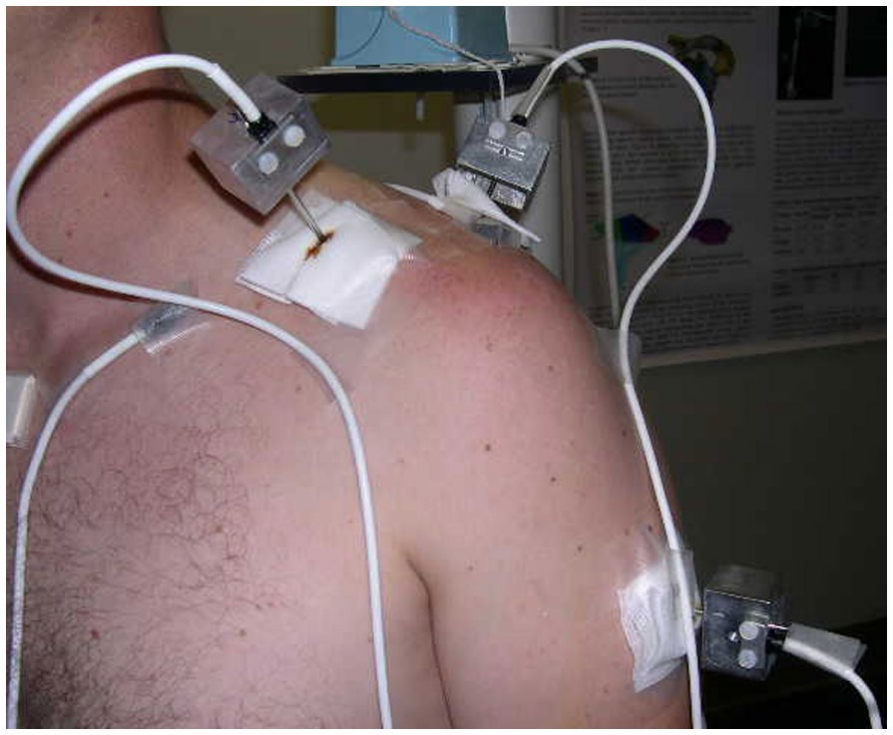
Intracortical pin placement in humerus and scapula in a representative subject.

Data collection for this study occurred at the time of data collection for a larger investigation (Ludewig et al. 2009; Lawrence, Braman, Laprade 2014; Lawrence, Braman, Staker, et al. 2014). As previously reported, (Braman et al. 2009; Ludewig et al. 2009) transcortical 2.5 mm pins were inserted with the use of a local anesthetic to the skin, subcutaneous tissue, and periosteum. Under sterile conditions and with fluoroscopic guidance the pins were placed into the humerus and scapula by an orthopedic surgeon (Figure 1). The insertion sites were between 1 and 2 cm in length to allow the pins to move freely during movement without interference by the skin. Sensors were then rigidly attached to the pins. Tracking pins placed in the humerus and scapula did not hinder hand placement for laxity test performance. A third EM sensor was secured by tape over the sternum to track trunk position. Glenohumeral translation values were collected for Anterior Drawer, Posterior Drawer, and Sulcus. A board certified, fellowship-trained shoulder surgeon performed all tests (EX1). Additionally, a physical therapist with expertise in clinical shoulder examination and biomechanics (EX2) performed the tests. This allowed assessment of inter-examiner kinematic repeatability across two clinical disciplines. No intra-examiner comparisons were performed because of the number of tests subjects underwent as part of the larger study.

The Anterior Drawer maneuver was performed as described by Silliman and Hawkins (Silliman and Hawkins 1993). Standing behind the subject, the examiner stabilized the scapula with their contralateral hand while applying a compressive, centralizing force into the glenoid followed by an anterior gliding force on the posterior humerus by the ipsilateral hand. Posterior Drawer was performed similarly, but with the gliding force directed posteriorly on the anterior humerus. Sulcus testing was performed with the subject’s arm at their side in neutral rotation. The examiner applied a longitudinally directed traction force by grasping the humeral epicondyles (Hawkins and Mohtadi 1991). If any examiner, for any test, performed two repetitions only the first repetition of a test was utilized for inter-examiner comparisons. The Anterior/Posterior test grade was judged by EX1 on a scale of 0–3 for each trial according to Hawkins and Mohtadi (1991). Sulcus test subjective grading is based on perceived translation distance (Altchek et al. 1990). Less than 1.0 cm perceived translation is defined as grade 1, 1–2 cm is defined as grade 2, and greater than 2 cm translation is a grade 3. Testing was performed in a sequential order of Anterior Drawer, Posterior Drawer, and Sulcus test. Only examiner EX1 provided laxity grades. Examiner EX2 was blinded to grades provided by EX1. Additionally, self-reported pain ratings on a visual analog scale (0–10) were measured with each test. If pain was verbalized, the examiner asked if the pain was shoulder joint pain or related to the transcortical pin.

### Data reduction

Anatomical landmarks were palpated, digitized and used to create embedded coordinate systems according to the International Society of Biomechanics (Wu et al. 2005). As previously described (Ludewig et al. 2009), for the scapula, the posterior acromioclavicular (AC) joint was digitized instead of the posterolateral acromion and the center of the humeral head was located using a functional, pivot center method as described by An et al. (1990). For the purpose of defining initial and final positions of the test movement, scapulothoracic angular motion was utilized. The sensors detect some scapular movement during the tests with only one hand to stabilize the scapula and the other imposing the test motion, as performed in clinical practice. This scapular motion was included to avoid defining the humeral movement by the dependent variable (humeral head translation). Angular motion was described using Euler angles (Wu et al. 2005). The time point at which the maximum position of the scapula had been reached defined the final position of the test movement and the rest position defined the start position. Subtraction of the start position at rest from the final position of the humeral head provided the time points for calculating the humeral head translation vector displacement for each plane of interest. Humeral translations were described as vector displacement values of the center of the humeral head relative to the origin of the scapula coordinate system.

### Statistical analysis

#### Inter-examiner test kinematic repeatability

Statistical analyses were performed utilizing IBM SPSS Statistics for Macintosh, Version 24 (Armonk, NY: IBM Corp). Intraclass correlation coefficients (Type 2,1) were performed to check inter-examiner kinematic repeatability of humeral translations for each laxity test (Fleiss 1986). To quantify error in the same units of measurement as the tests, the standard error of the measurement was calculated as the square of the mean square error term from a one-way ANOVA table with subjects as the factor (Stratford and Goldsmith 1997). Additionally, the mean absolute difference of translation magnitude, and a paired *t*-test were calculated between examiners for each laxity test.

#### Relationship between single test subjective grades and joint translations

Linear relationships were examined with regression analyses of EX1’s grade to the glenohumeral translation for each clinical test. Potential outliers were identified with residual plots and studentized residual calculations. Additionally, descriptive analyses were performed to identify the median and range of translation for each laxity test subjective grade.

#### Relationship of overall glenohumeral laxity to a composite subjective score

To examine the relationship of overall glenohumeral laxity for each subject to their subjective grades, two composite variables were calculated. A composite subjective laxity score was calculated from the mean of EX1’s grades during the laxity testing for each subject. To calculate the overall glenohumeral laxity for each subject, a root mean square (RMS) calculation was performed which involved squaring the translation values from each test, averaging the squared values, then taking the square root to convert back to original magnitudes. A linear regression analysis was then performed with the composite subjective score set as the predictor variable and the overall joint laxity set as the response variable in the analysis. Presence of any overly influential values was checked with Cook’s D (Cook 1979). No Cook’s D values were >1, indicating no influential outliers, and thus no data points were excluded from the model. The a priori alpha level was set at 0.05.

## Results

The translation magnitude for each subject, test, and examiner has been provided as supplementary material (Supplementary Table 1). Subjective reports of pain on the visual analog scale during any of the test maneuvers averaged less than 1.3 for pain attributed by the subjects to either the joint or the transcortical pins. Inter-examiner kinematic repeatability of exam translations is summarized in Table 3. A valid ICC for the Posterior Drawer test could not be calculated because the between subject variance was too small (Fleiss 1986). The standard error of the measurement (SEM) for the Posterior Drawer was 2.6 mm. The ICC for the Anterior Drawer was 0.87 (95% confidence interval 0.62–0.96; SEM = 1.0 mm) and for the Sulcus test was 0.84 (95% confidence interval 0.51–0.95, SEM = 1.2 mm). Paired *t*-tests demonstrated significantly less mean translation by EX1 for the Anterior Drawer test compared to EX2 (Anterior Drawer 0.9 mm, *p* < 0.05) and significantly more translation by EX1 for the Posterior Drawer test (2.2 mm, *p* < 0.05). There was no significant difference in translations for the Sulcus tests. The mean absolute difference in translations for Anterior Drawer was 1.3 mm (SD = 0.7 mm), Posterior Drawer 2.6 mm (SD = 2.6 mm), and Sulcus Tests was 1.8 mm (SD = 1.4 mm).

**Table 3.** Descriptive values and inter-examiner translation repeatability for shoulder laxity tests.

Test	Anterior drawer		Posterior drawer		Sulcus	
Examiner	EX1	EX2	EX1	EX2	EX1	EX2
Mean Translation (mm)	3.1	4.0	−3.9	−1.7	−3.0	−2.9
ICC (CI)	0.87 (0.62–0.96)		na		0.84 (0.51–0.95)	
SEM (mm)	1.0		2.6		1.2	

Note:

ICC, intraclass correlation coefficient (2,1); CI, 95% confidence interval; *na*, not applicable (ICC calculation for posterior drawer not valid due to low between-subject variation (Fleiss 1986); SEM, standard error of measurement.

**Figure 2.**
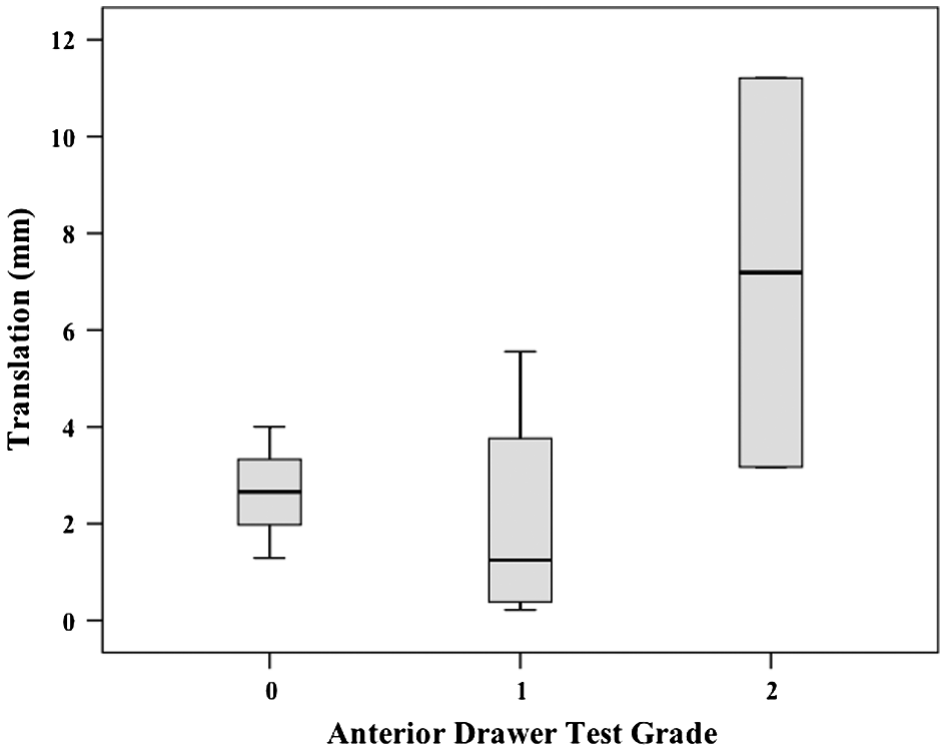
Box plot (median, interquartile range, minimum and maximum) of EX1 translations for each subjective grade during Anterior Drawer testing.

**Figure 3.**
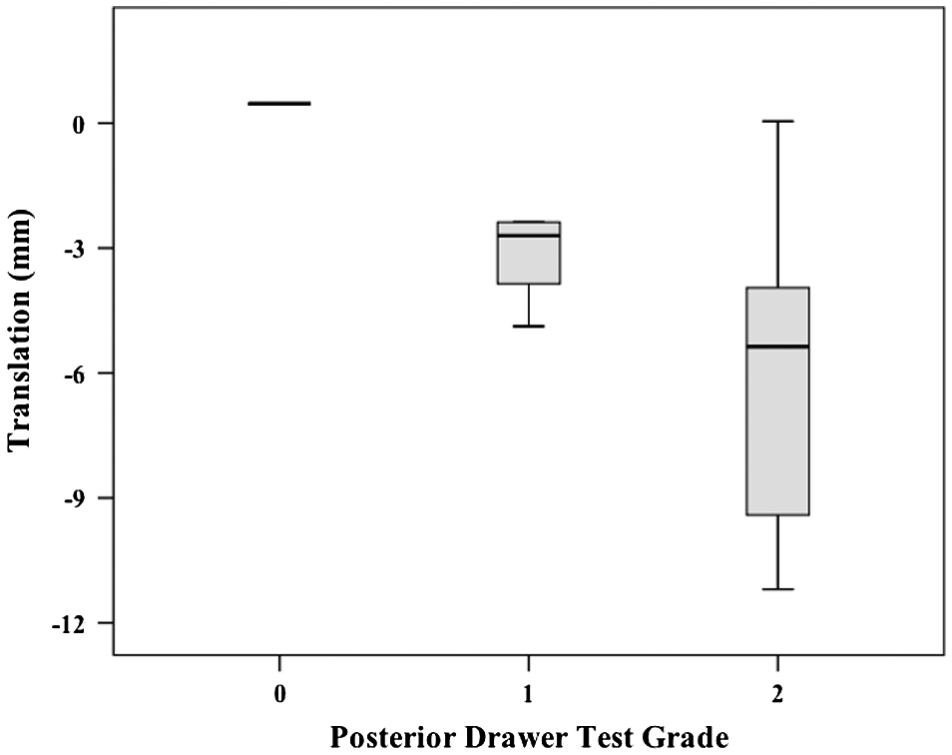
Box plot (median, interquartile range, minimum and maximum) of EX1 translations for each subjective grade during Posterior Drawer testing.

Note: A single line without surrounding box plots indicates only one observation at that grade level.

**Figure 4.**
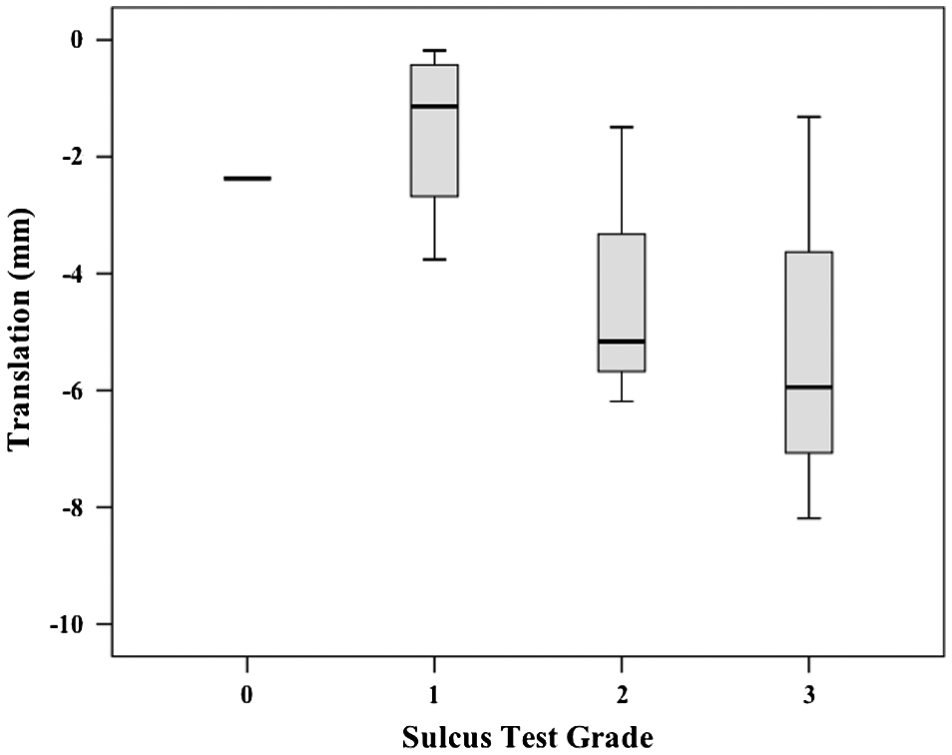
Box plot (median, interquartile range, minimum and maximum) of EX1 translations for each subjective grade during Sulcus testing.

Note: A single line without surrounding box plots indicates only one observation at that grade level.

No linear relationships were demonstrated between the subjective scores for any single laxity test and translations. The *r*^2^ values ranged from 0.19 to 0.33 and were all non-significant. Descriptively, a general trend was observed that the smallest median translations corresponded to lowest grades and largest median translations corresponded to highest grades across the laxity tests (Figures 2–4).

**Figure 5.**
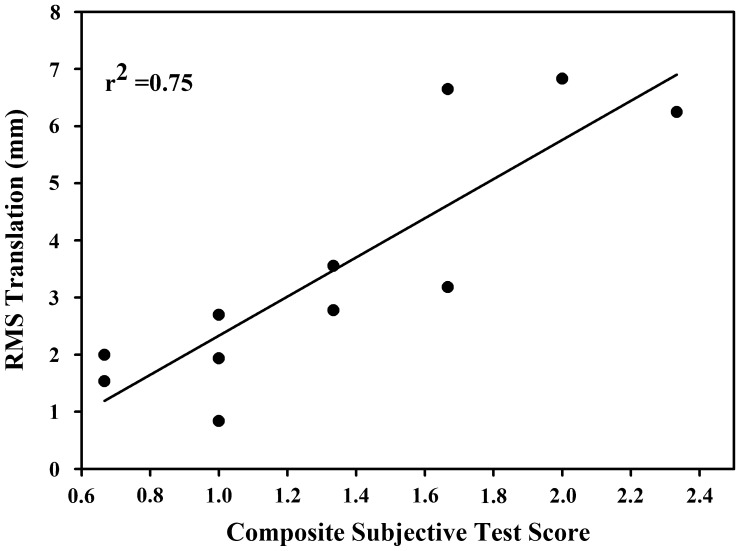
Regression of combined Anterior Drawer, Posterior Drawer and Sulcus tests RMS translation with the composite subjective test score.

Note: *r*^2^ = 0.75, *r* = 0.86, *p* < 0.005.

The simple linear regression analysis comparing composite subjective scores and overall laxity from the RMS calculation of all three instability tests (Anterior Drawer, Posterior Drawer, and Sulcus tests) revealed a significant association (*r*^2^ = 0.75, *r* = 0.86, *p* < 0.005) (Figure 5).

## Discussion

Our results demonstrated good kinematic repeatability between examiners on two of three laxity tests (Anterior Drawer and Sulcus tests). Although subjective scores from individual tests were not associated with their test-specific translation grades, taken together, the composite subjective score from the Anterior and Posterior Drawer and Sulcus tests was highly associated with overall glenohumeral joint laxity (*r*^2^ = 0.75).

### Inter-examiner test kinematic repeatability

Our study differed considerably in how reliability of translation was assessed from prior work. Sauers et al. (2001) assessed the repeatability of the magnitude of the applied loads during mechanically constrained laxity tests. Understanding how applied joint loading may affect stress/strain characteristics of the joint does not assess how differences in examiner’s subjective grades may be influenced by differences in the amount of translation examiners are imparting to the joint. Therefore, studies assessing clinical laxity test reliability must directly evaluate whether examiners reproduce the same amount of translation during the examination. A study by Lippit et al. (1994) used bone fixed motion tracking but only for a single examiner’s performance. They reported ‘highly reproducible’ trial-to-trial translation kinematics in both magnitude and direction for three repetitions.

Most studies describe the reliability of laxity tests subjective grades based on the agreement between examiners. These studies have demonstrated poor overall agreement in subjective grades of translations between examiners (Levy et al. 1999; Ellenbecker et al. 2002; Hegedus et al. 2008; May et al. 2010). However, our examiners demonstrated good (Portney and Watkins 2000) between-examiner kinematic repeatability of translations occurring during the Anterior Drawer and Sulcus tests (ICC = 0.84 and 0.87). The subjective nature of the laxity test grading systems likely contributes to the limited between-examiner agreement despite the possibility examiners are producing similar joint translations during the tests.

Furthermore, our study suggests the grading system for a single test may be ‘offset’ relative to underlying bone translations. For example, the average translation displacements of the humeral head center recorded during the Sulcus test results were approximately 3 mm, but the subjective grading system is based on centimeter increments. Similarly, Anterior and Posterior Drawer tests have been based on percentage of humeral diameter displacement (Hawkins and Mohtadi 1991) and if an average humeral head diameter of 46 mm (Boileau and Walch 1997) is considered, translations of potentially greater than 23 mm are being perceived. The examiner may perceive these large magnitudes of motion, however, less translatory motion is likely occurring at the joint. Other studies utilizing radiography and ultrasound measurements of joint translation have demonstrated similar translations of typically less than 7 mm with varying joint loads and patient populations (Ellenbecker et al. 2000; Borsa et al. 2001; Borsa, Scibek, et al. 2005; Borsa, Wilk, et al. 2005; Cheng et al. 2008). Therefore, the grading systems appear to represent an examiner’s interpretation of imparted glenohumeral motion, not the actual magnitude of translation occurring.

There were no constraints on the imposed translation force and no pre-study training other than verbal agreement between the two examiners. Therefore, the finding of good translation repeatability in two laxity tests suggests that despite poor inter-examiner reliability of subjective grades in the literature, these tests remain clinically relevant. Inconsistencies in the magnitude of force application and disparate examiner training have been considered as potential causes of low subjective grade reliability observed in laxity tests (Levy et al. 1999; Sauers et al. 2001). Our findings suggest it is possible to produce similar clinical laxity tests kinematics by two examiners with different clinical backgrounds. Further study involving more clinicians with diverse training is necessary to confirm the repeatability of laxity test kinematics. The poor repeatability observed in the Posterior Drawer test may have been caused by an inconsistency in achieving the initial neutral position between the examiners. Follow up debriefing revealed that the joint compression step prior to the posterior glide might have been applied inconsistently between the examiners. This may explain the differences in translations imparted by the examiners for the Anterior Drawer (0.9 mm) and Posterior Drawer (2.2 mm).

Additional work is needed to develop methods to improve correlation of subjective grading with actual translation. The development of more objective tools to provide measures in a clinically feasible manner may also be beneficial (Borsa et al. 2001; Sauers et al. 2001; Sein et al. 2008). However, the clinical implementation of complex mechanical devices is likely to be limited. Furthermore, the good inter-examiner kinematic repeatability in this study suggests that joint loading devices designed to impose consistent forces across the joint may not be necessary. This study provides initial evidence that the tests provide potentially useful information but more valid and reliable techniques to measure translations occurring during clinical laxity tests is important to enhance their utility. Emerging technology in clinical sensors and imaging approaches may assist in this effort.

### Relationship of subjective test grades to joint laxity

Laxity tests are routinely utilized in the clinic despite documented poor inter-examiner reliability and no studies of translation grading validity (Levy et al. 1999; Ellenbecker et al. 2002; May et al. 2010; Hegedus et al. 2012). However, clinicians must still make treatment decisions incorporating information from these laxity tests when better alternatives do not exist. How best to incorporate the finding from an isolated test to assess joint laxity in cases of subtle, ‘microinstabilities’ is not currently known. Individual test grades in this study were not linearly associated with translation. Regression can be applied to ordinal data, but our individual test data did not fit a regression line well. Subsequently, descriptive trends were observed demonstrating that lower grades had smaller median translations and vice versa. However, the ranges of translations at each grade were large (2.7–11.2 mm). This result indicates that a single laxity test grade may not provide the precision necessary to sufficiently overcome variation at each grade level to diagnose microinstability or glenohumeral hypomobility.

Although individual test precision may be limited, this study provides a biomechanically supported approach for interpreting these three laxity tests together to more precisely predict joint laxity. In doing so, clinicians may have the potential to clinically identify subtle differences in overall joint laxity between patients. Although clustering signs and symptoms to provide diagnostic guidance is not uncommon (Wainner et al. 2003; Walsworth et al. 2008; Michener et al. 2009) this study is the first, to our knowledge, that utilizes composite subjective scores from three tests to provide an overall assessment of joint laxity. When combined, the potential clinical utility of the three tests was substantially improved (*r*^2^ = 0.75, *p*< 0.005). The ability to clinically identify a continuum of shoulder joint laxity permits subgrouping of patients. In turn, targeted treatment interventions for individuals can be developed and studied. Theoretically, individuals scoring low on the scale (glenohumeral hypomobility) would benefit from interventions designed to improve joint motion and conversely individuals with high scores would benefit from joint stabilization techniques.

Interpretation of this study’s results should be considered in light of its limitations. The small sample size may impact the distribution of translation magnitudes. Although minor skewness and kurtosis existed, no statistically influential data points or outliers were detected in follow-up tests. The small sample size potentially limits generalizability of its findings beyond this subject population.

The inclusion criteria for this study were not created to identify subjects with shoulder instability. Rather, they were developed to represent the heterogeneous group of patients commonly seen in the clinic with shoulder pain. These clinical laxity tests are frequently used to diagnose microinstability theorized to contribute to shoulder dysfunction (Bak and Fauno 1997; McMaster et al. 1998). It was our goal to specifically determine the utility of these clinical tests in a population without definitive instability related to joint dislocations.

Generalizability of this study may be affected by the persistent nature of atraumatic shoulder pain of the study’s subjects (average of 9 year history of intermittent shoulder complaints). However, many patients with overuse conditions only seek clinical care after repeated bouts of symptoms. Therefore, these subjects represent the clinical population seen for recurrent, non-acute shoulder symptoms. The atraumatic and persistent nature of subjects’ symptoms are supportive of the common clinical theory that increased laxity played a causative role in subject’s development of shoulder pain. However, no conclusions regarding casual relationships can be made from this study because of the cross-sectional design.

The invasive nature of the study limited data collection time and therefore only inter-examiner repeatability was examined. However, previous investigators have demonstrated low intra-examiner variance utilizing similar testing methods (Harryman et al. 1992). Additionally, our results demonstrated high inter-examiner repeatability. Because intra-examiner repeatability is typically greater than inter-examiner repeatability, we believe our examiners would have demonstrated similarly high inter-examiner repeatability.

Non-invasive, imaging-based technology such as ultrasonography that is minimally affected by skin motion could allow simultaneous joint motion measurement during movement but their two-dimensional nature limits accuracy. Studies examining clinical test kinematics utilizing developing techniques matching radiographic images of joint movement with 3D bone anatomy (2D/3D shape matching) hold promise for improving accuracy and decreasing the necessity of invasive tracking methods.

This study suggests laxity test translations are reproducible, and when findings from multiple tests are taken together, composite subjective scores may improve precision in identifying the level of joint laxity in patients with shoulder pain. The ability to clinically identify subtle differences in movement abnormalities in patients is an important step in developing targeted, biomechanically sound interventions.

## Conclusion

Clinicians with differing training performing shoulder laxity tests have the potential to demonstrate high between-examiner kinematic repeatability. The composite subjective scores from Anterior Drawer, Posterior Drawer, and Sulcus tests were strongly associated with overall joint laxity.

## Disclosure statement

We affirm that we have no financial affiliation (including research funding) or involvement with any commercial organization that has a direct financial interest in any matter included in this manuscript, except as disclosed in an attachment and cited in the manuscript. Any other conflict of interest (i.e. personal associations or involvement as a director, officer, or expert witness) is also disclosed in an attachment.

## Funding

The project was supported by the National Institutes of Health (NIH) under grant number [K01HD042491] (Ludewig) from the Eunice Kennedy Shriver National Institutes of Child Health and Human Development. The content is solely the responsibility of the authors and does not necessarily represent the official views of the Eunice Kennedy Shriver National Institutes of Child Health and Human Development or the National Institutes of Health.

## Supplemental data

Supplemental data for this article can be accessed at https://doi.org/10.1080/23335432.2017.1372217

## Supplementary Material

TBBE_1372217_Supplementary_material.docx
