# Supplementary material for: Three-dimensional kinematics of shoulder laxity examination and the relationship to clinical interpretation
Source: Int Biomech. 2017 Dec 15;4(2):77–85. doi: 10.1080/23335432.2017.1372217 (PMC7857453; doi:10.1080/23335432.2017.1372217)
Supplement: TBBE_1372217_Supplementary_material.docx [file TBBE_A_1372217_SM3159.docx]

| Supplementary TABLE 1. Translation Magnitudes for Each Test and Examiner | | | | | | | | | |
| --- | --- | --- | --- | --- | --- | --- | --- | --- | --- |
|  | Anterior Drawer (mm) | | | Posterior Drawer (mm) | | | Sulcus Test (mm) | | |
| Subject | EX1 (grade) | EX2 | Abs. Diff. | EX1 (grade) | EX2 | Abs Diff. | EX1 (grade) | EX2 | Abs. Diff. |
| 1 | 1.0 (1) | 2.7 | 1.7 | -4.0 (2) | -1.3 | 2.7 | -3.8 (1) | -4.5 | 0.7 |
| 2 | 2.7 (0) | 1.5 | 1.2 | -2.4 (1) | -1.2 | 1.2 | -1.5 (2) | -0.5 | 1.0 |
| 3 | 0.4 (1) | 1.7 | 1.3 | 0.3 (1) | 0.6 | 0.3 | -1.6 (1) | -0.4 | 1.2 |
| 4 | 1.3 (0) | 3.4 | 2.1 | -2.7 (1) | -1.0 | 1.7 | -0.7 (1) | -1.2 | 0.5 |
| 5 | 3.2 (2) | 4.1 | 1.0 | 0.5 (0) | -1.4 | 1.8 | -2.4 (0) | -0.1 | 2.3 |
| 6 | 11.2 (2) | 10.0 | 1.2 | -5.4 (2) | -5.5 | 0.1 | -1.3 (3) | -2.3 | 1.0 |
| 7 | 4.0 (0) | 6.0 | 2.0 | -11.2 (2) | -3.0 | 8.2 | -5.9 (3) | -7.1 | 1.2 |
| 8 | 5.6 (1) | 5.7 | 0.2 | -9.4 (2) | -2.3 | 7.1 | -8.2 (3) | -10.4 | 2.2 |
| 9 | 3.8 (1) | 3.9 | 0.1 | -3.9 (1) | -2.4 | 1.4 | -0.2 (1) | -2.3 | 2.1 |
| 10 | 1.5 (1) | 3.6 | 2.1 | 0.0 (2) | -0.2 | 0.2 | -6.2 (2) | -4.1 | 2.1 |
| 11 | 0.2(1) | 1.5 | 1.3 | -4.9 (1) | -1.6 | 3.3 | -5.2 (2) | 0.6 | 5.7 |
|  | Mean (SD) | | 1.3 (0.7) | Mean (SD) | | 2.6 (2.6) | Mean (SD) | | 1.8 (1.4) |
| Grade, examiner EX1 subjective grade; Abs. Diff., absolute difference in translation magnitude between examiners. Means are mean absolute differences in translation magnitudes | | | | | | | | | |
